# Clinical profile of patients with *ATP1A3* mutations in Alternating Hemiplegia of Childhood—a study of 155 patients

**DOI:** 10.1186/s13023-015-0335-5

**Published:** 2015-09-26

**Authors:** Eleni Panagiotakaki, Elisa De Grandis, Michela Stagnaro, Erin L. Heinzen, Carmen Fons, Sanjay Sisodiya, Boukje de Vries, Christophe Goubau, Sarah Weckhuysen, David Kemlink, Ingrid Scheffer, Gaëtan Lesca, Muriel Rabilloud, Amna Klich, Alia Ramirez-Camacho, Adriana Ulate-Campos, Jaume Campistol, Melania Giannotta, Marie-Laure Moutard, Diane Doummar, Cecile Hubsch-Bonneaud, Fatima Jaffer, Helen Cross, Fiorella Gurrieri, Danilo Tiziano, Sona Nevsimalova, Sophie Nicole, Brian Neville, Arn M. J. M. van den Maagdenberg, Mohamad Mikati, David B. Goldstein, Rosaria Vavassori, Alexis Arzimanoglou, The Italian IBAHC Consortium, The French AHC Consortium, The International AHC Consortium

**Affiliations:** Epilepsy, Sleep and Pediatric Neurophysiology Department (ESEFNP), University Hospitals of Lyon (HCL), Lyon, France; Department of Child Neuropsychiatry, G. Gaslini Hospital, University of Genoa, Genoa, Italy; Center for Human Genome Variation, Duke University School of Medicine, Durham, NC USA; Department of Medicine, Duke University School of Medicine, Durham, NC USA; Department of Child Neurology, Sant Joan de Déu Hospital, Barcelona, Spain; Department of Clinical and Experimental Epilepsy, University College London Institute of Neurology, London, UK; Department of Human Genetics, Leiden University Medical Centre, Leiden, The Netherlands; Department of Child Neurology, University Hospitals Leuven, Leuven, Belgium; Department of Molecular Genetics, Neurogenetics Group, VIB, Antwerp, Belgium; Department of Neurology, Charles University, First Faculty of Medicine and Teaching Hospital, Prague, Czech Republic; Department of Medicine, University of Melbourne, Austin Health, Melbourne, Australia; Department of Paediatrics, University of Melbourne, Royal Children’s Hospital, Melbourne, Australia; Department of Genetics, University Hospitals of Lyon (HCL) and Claude Bernard Lyon I University, Lyon, France; Lyon Neuroscience Research Center (CRNL), CNRS UMR 5292, INSERM U1028, Lyon, France; Biostatistics Department, University Hospitals of Lyon and UMR 5558, Lyon, France; Child Neurology Unit, Maggiore Hospital, Bologna, Italy; Department of Child Neurology, Armand Trousseau Hospital, APHP, Paris, France; Department of Neurology, Pitié-Salpêtrière Hospital, APHP, Paris, France; Institute of Child Health, University College London, London, UK; Institute of Medical Genetics, University Cattolica del Sacro Cuore, Policlinics A. Gemelli, Rome, Italy; Institut National de la Santé et de la Recherche Médicale, U975, Centre de Recherche de l’Institut du Cerveau et de la Moelle, Paris, France; Centre National de la Recherche Scientifique, UMR7225, Paris, France; Department of Neurology, Leiden University Medical Centre, Leiden, The Netherlands; Division of Pediatric Neurology and Department of Neurobiology, Duke University, School of Medicine, Durham, NC USA; Associazione Italiana per la Sindrome di Emiplegia Alternante (A.I.S.EA Onlus), Lecco, Italy; DYCOG team, Lyon Neuroscience Research Centre (CRNL), INSERM U1028; CNRS UMR 5292, Lyon, France

**Keywords:** Alternating hemiplegia of childhood, *ATP1A3*, Genotype-phenotype

## Abstract

**Background:**

Mutations in the gene *ATP1A3* have recently been identified to be prevalent in patients with alternating hemiplegia of childhood (AHC2). Based on a large series of patients with AHC, we set out to identify the spectrum of different mutations within the *ATP1A3* gene and further establish any correlation with phenotype.

**Methods:**

Clinical data from an international cohort of 155 AHC patients (84 females, 71 males; between 3 months and 52 years) were gathered using a specifically formulated questionnaire and analysed relative to the mutational *ATP1A3* gene data for each patient.

**Results:**

In total, 34 different *ATP1A3* mutations were detected in 85 % (132/155) patients, seven of which were novel. In general, mutations were found to cluster into five different regions. The most frequent mutations included: p.Asp801Asn (43 %; 57/132), p.Glu815Lys (16 %; 22/132), and p.Gly947Arg (11 %; 15/132). Of these, p.Glu815Lys was associated with a severe phenotype, with more severe intellectual and motor disability. p.Asp801Asn appeared to confer a milder phenotypic expression, and p.Gly947Arg appeared to correlate with the most favourable prognosis, compared to the other two frequent mutations. Overall, the comparison of the clinical profiles suggested a gradient of severity between the three major mutations with differences in intellectual (*p* = 0.029) and motor (*p* = 0.039) disabilities being statistically significant. For patients with epilepsy, age at onset of seizures was earlier for patients with either p.Glu815Lys or p.Gly947Arg mutation, compared to those with p.Asp801Asn mutation (*p* < 0.001). With regards to the five mutation clusters, some clusters appeared to correlate with certain clinical phenotypes. No statistically significant clinical correlations were found between patients with and without *ATP1A3* mutations.

**Conclusions:**

Our results, demonstrate a highly variable clinical phenotype in patients with AHC2 that correlates with certain mutations and possibly clusters within the *ATP1A*3 gene. Our description of the clinical profile of patients with the most frequent mutations and the clinical picture of those with less common mutations confirms the results from previous studies, and further expands the spectrum of genotype-phenotype correlations. Our results may be useful to confirm diagnosis and may influence decisions to ensure appropriate early medical intervention in patients with AHC. They provide a stronger basis for the constitution of more homogeneous groups to be included in clinical trials.

**Electronic supplementary material:**

The online version of this article (doi:10.1186/s13023-015-0335-5) contains supplementary material, which is available to authorized users.

## Background

Alternating hemiplegia of childhood (AHC) is a rare neurological disorder characterized by transient episodes of alternating hemiplegia/hemiparesis, dystonic attacks, paroxysmal abnormal ocular movements, epileptic seizures and episodes of autonomic dysfunction [1–3]. The disease usually starts before 18 months of life and in the majority of patients before the age of 6 months. Plegic and tonic attacks disappear with sleep [4, 5]. Between attacks patients have an abnormal neurological examination often presenting ataxia, dystonia and other involuntary abnormal movements, and almost all present an intellectual disability [6, 7]. AHC has a prevalence of 1:100,000 children [8]. Our previous results emphasized the significant variability of the disease course between individuals and indicated no general pattern of progression [9].

Mutations have been identified in some AHC patients in the following genes: *CACNA1A* [10], *SLC1A3* [11], *SLC2A1* [12, 13], and *ATP1A2* (AHC1, MIM number 104290) [14, 15]. The majority of these cases were atypical with features overlapping with either familial or non-familial hemiplegic migraine. Further studies in larger numbers of patients have failed to confirm a correlation between mutations in these genes and alternating hemiplegia of childhood [5, 9, 16–20].

In 2012, mutations in the *ATP1A3* gene (MIM 182350), located at 19q13.2 [hg19], were identified as the primary cause of AHC [21–23] (AHC2, MIM 614820). Mutations in *ATP1A3* are found in approximately 75 % of cases and the disease is transmitted as an autosomal dominant trait. The mutations are usually *de novo*, but some have been found to be transmitted to offspring [21]. The *ATP1A3* gene (23 exons, ORF contains 3042 base-pairs) encodes the sodium-potassium (Na+/K+) ATPase α3 subunit (1014 amino acids) that contains 6 cytoplasmic, 10 helical and 5 extracellular domains. Mutations in the *ATP1A3* gene, are also found in patients with dystonia 12 (rapid-onset dystonia parkinsonism; RDP, MIM 128235) [24–27] and CAPOS (cerebellar ataxia, areflexia, pes cavus, optic atrophy and sensorineural hearing loss, MIM 601338) syndrome [28]. RDP is a non-dopa-responsive dystonia, with rapid onset of a few minutes to a few days before stabilization. The age at onset is between 9 months [29] and 59 years and triggering factors are physical (e.g. exercise or childbirth) or psychological stress. CAPOS syndrome is characterized by an early-childhood onset of recurrent episodes of acute ataxia associated with febrile illnesses. These acute episodes tend to decrease with time, but the neurologic sequelae are permanent and progressive, resulting in gait and limb ataxia and areflexia. Affected individuals also develop progressive visual impairment due to optic atrophy and sensorineural hearing loss beginning in childhood [28]. With the addition of our data, no less than 83 *ATP1A3* mutations have been described in patients with these three disorders [30–41] (Additional file 1).

The present study describes data obtained from a large international cohort, which is, in part, based on the initial European web-based registries ENRAH (European Network for Research on Alternating Hemiplegia) [42] and nEUroped (European Network on Rare Paediatric Neurological Diseases) [43]. The aim was to identify possible correlations between clinical phenotype and different *ATP1A3* gene mutations. In addition, the phenotypes of patients with and without *ATP1A3* mutations were also compared.

## Methods

This work was based on the efforts of the *International Consortium for the Research on AHC* (IAHCRC [44]) formed in 2012 after the identification of mutations in *ATP1A3* in AHC patients. The group involves clinicians, geneticists and researchers from Europe, USA and Australia and works in close collaboration with patient organizations, most of whom had already participated in the ENRAH and nEUroped projects.

An AHC patient database was formed within the framework of these two projects, in which clinical data are continuously being updated. The medical data reported here were centralised from nine different countries: France (57 patients), Italy (41), Spain (16), United Kingdom (10), USA (8), The Netherlands (7), Belgium (7), Czech Republic (5) and Australia (4).

### Inclusion criteria

Diagnosis of AHC was based on Aicardi’s criteria, as previously reported [4, 9]: (1) onset of paroxysmal events before 18 months of age; (2) repeated bouts of hemiplegia involving the right and left side of the body during some attacks; (3) episodes of bilateral hemiplegia or quadriplegia starting either as generalization of a hemiplegic episode or bilateral from the start; (4) other paroxysmal disturbances including tonic/dystonic attacks, nystagmus, strabismus, dyspnoea and other autonomic phenomena occurring during hemiplegic bouts or in isolation; (5) immediate disappearance of all symptoms upon sleep, with probable recurrence of long-lasting bouts, 10–20 min after awakening; (6) evidence of developmental delay, intellectual disability, neurological abnormalities, choreoathetosis and dystonia or ataxia; and (7) not attributable to other disorders.

### Phenotypic data—questionnaire

To assess clinical phenotype, a questionnaire was designed (see Additional file 2).

Information was related to various time points or epochs: first, *lifetime information* concerning different signs and symptoms appearing at least once over a lifetime; second, *time at inclusion in the database*; and third, the *time period between 6 and 12 years old. Lifetime information* allowed us to investigate whether a sign/symptom was present previously, even if it was no longer present at the time of inclusion or at 6–12 years old. The time period of 6–12 years old was used in order to be able to compare data at a similar age, as subjects included had very different ages.

Details concerning paroxysmal and non-paroxysmal features were collected for all age epochs. For plegic and tonic attacks, the following details were noted: semiology, frequency, length and triggering events. The occurrence of an epileptic seizure, in contrast to other paroxysmal events, was considered when either the semiology of the event was definitively indicative, interictal EEG changes corroborated the clinical observations, or an epileptic event was confirmed by EEG.

Intellectual disability was categorized as “mild” (IQ of 50–69), “moderate” (IQ of 35–49), or “severe” (IQ less than 35). The questionnaire completed by the clinicians was based either on IQ tests, when available, or indirect estimation of the degree of intellectual disability from clinical description and information regarding educational placement and/or professional integration in adulthood.

### Data collection

Data collection was undertaken by the delegated participating centre managers (one per reference centre), who completed the questionnaire either after direct contact with patients and/or after revision of medical records, using additional information provided by the treating physician (paediatric neurologist or neurologist) or family. National parent associations assisted in the collection of data.

Research was conducted in accordance with the Declaration of Helsinki, and all procedures were carried out with the adequate understanding and written consent of the subjects or their parents, according to the appropriate national ethical committees, in accordance with national legislation and regulations.

### Mutation analysis

DNA was extracted from blood, saliva, or buccal specimens from the probands and parents using standard procedures. The 23 exons and immediately flanking splice sites were Sanger sequenced in proband DNA using the primers listed in Additional file 3. Technical details of methods were reported in our previous, primary publication [21]. The mutation analysis was extended wherever possible to the parents to define if the mutation was *de novo. ATP1A3* mutations were considered as probably pathogenic if they had occurred *de novo* and if the prediction tools were in favour of a deleterious effect. To ensure no patients were analysed more than once in this study, patients with the same rare *ATP1A3* mutation, or lack thereof, were first assessed where possible for identical dates of birth and gender. In cases where this approach could not be taken based on site specific patient confidentially rules, concordant patients were genotyped at a series of 13 polymorphic sites in the genome (Additional file 4) to establish all patients studied were unique.

SIFT, Polyphen-2 and Mutation Taster were used for *in silico* prediction of pathogenicity of the missense mutations.

Reference sequences for corresponding *ATP1A3* transcript and protein were [NM_152296.3] and [Uniprot P13637], respectively.

Analysis of RNA processing was not performed in this study.

### Statistical analysis

Quantitative characteristics were described by the quartiles and the minimum and maximum values. Box plots were used to represent the distributions.

Qualitative characteristics were described by the absolute and relative frequencies in each category. Horizontal bar plots were used to represent the repartition of the patients in the different categories. Statistical comparisons were performed when groups of patients with the three most frequent mutations (p.Asp801Asn, p.Glu815Lys, or p.Gly947Arg) were compared, as well as between patients with and without any mutation. Comparisons were performed for the time period between 6 and 12 years.

The Kruskal-Wallis test and the Fisher exact test were used for quantitative and qualitative characteristics, respectively. In order to take into account the multiplicity of the tests, the type-I error was controlled using the approach of Benjamin and Yekutieli [45].

Analysis was carried out using the R software, version 3.1.0 (Free Software Foundation).

## Results and discussion

A total of 155 AHC patients (84 females and 71 males) were included. At inclusion, patients were aged between 3 months and 52 years.

Thirty-four different *ATP1A3* mutations were detected in 85 % (132/155) AHC patients. The most frequent were p.Asp801Asn (43 %, 57/132), p.Glu815Lys (16 %, 22/132) and p.Gly947Arg (12 with c.2839G > A and three with c.2839G > C) (11 %, 15/132). All patients with p.Gly947Arg were considered as a single group, regardless of the nucleotide substitution. Less frequent mutations were p.Gly755Ser (in three patients) and p.Ser137Tyr, p.Ser772Arg (one with c.2314A > C and one with c.2316C > G), p.Asn773Ser, p.Thr804Ile, p.Ser811Pro, p.Val919del and p.Asp923Asn (each present in two patients). There were 21 more mutations find each one at one patient.

Clinical characteristics of patients harbouring the three most common mutations (p.Asp801Asn, p.Glu815Lys and p.Gly947Arg) and patients with no *ATP1A3* mutation are shown in detail in the supplementary data (Additional file 5). For the period between 6 and 12 years, the age at which patient data was directly compared, information was available for 105 patients, and 63/105 (60 %) had one of the three most frequent mutations (38 with p.Asp801Asn, 14 with p.Glu815Lys and 11 with p.Gly947Arg).

### Genotype - phenotype correlations

**Fig. 1:**
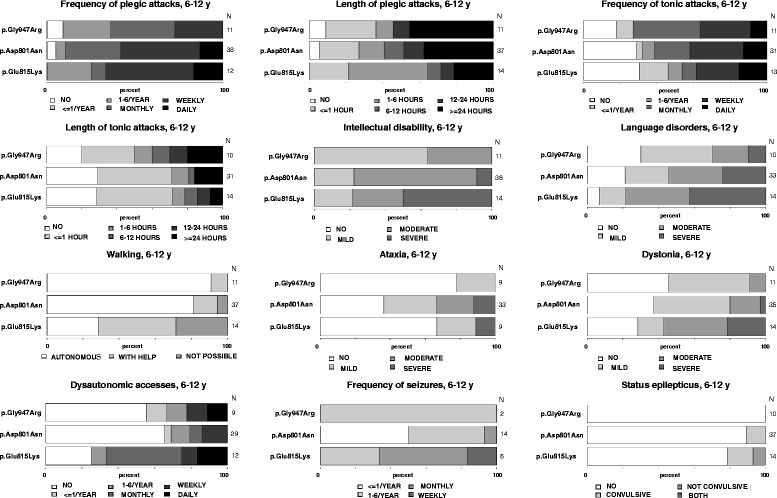
Clinical variables with their degrees of severity, concerning the three most frequent mutations. Degrees of severity and their gray scale code are presented on the bottom of each bar plot, whereas absolute number of patients on the right. Different degrees of severity are given in percentages and the 3 most frequent mutations are always presented with the p.Glu815Lys mutation on the bottom, the p.Asp801Asn mutation in the middle and the p.Gly947Arg on the top

A summary of clinical features and further patient information is presented in Fig. 1 and Additional file 5. *P* values are given only when differences were statistically significant.

As outlined in Fig. 1, comparison of the three most common mutations, p.Glu815Lys, p.Asp801Asn and p.Gly947Arg, revealed a gradient of severity of associated phenotypes. p.Glu815Lys was associated with the most severe phenotype, followed by p.Asp801Asn that appeared to confer a milder phenotypic expression, followed by p.Gly947Arg that correlated with the most favourable prognosis. The most pronounced differences regarding the severity of phenotypes between the three mutations were intellectual (*p* = 0.029) and motor (*p* = 0.039) disability, as well as age at onset of seizures which was earlier for patients with either p.Glu815Lys or p.Gly947Arg mutation, compared to those with p.Asp801Asn mutation (*p* < 0.001). In addition, there were also apparent trends in differences of severity regarding other aspects of the disease (language, dystonia, autonomic dysfunction, epilepsy) (Fig. 1) however, these were not statistically significant, possibly due to the small number of patients.

Differences in the length and frequency of (hemi)plegic and tonic attacks was, however, less obvious. A plausible explanation for this could be the retrospective nature of the determination of the precise frequency and duration of attacks in patients that were ambulatory relative to those who were bedridden in settings in which these features may not have been specifically investigated.

#### p.Glu815Lys mutation

**Fig. 2:**
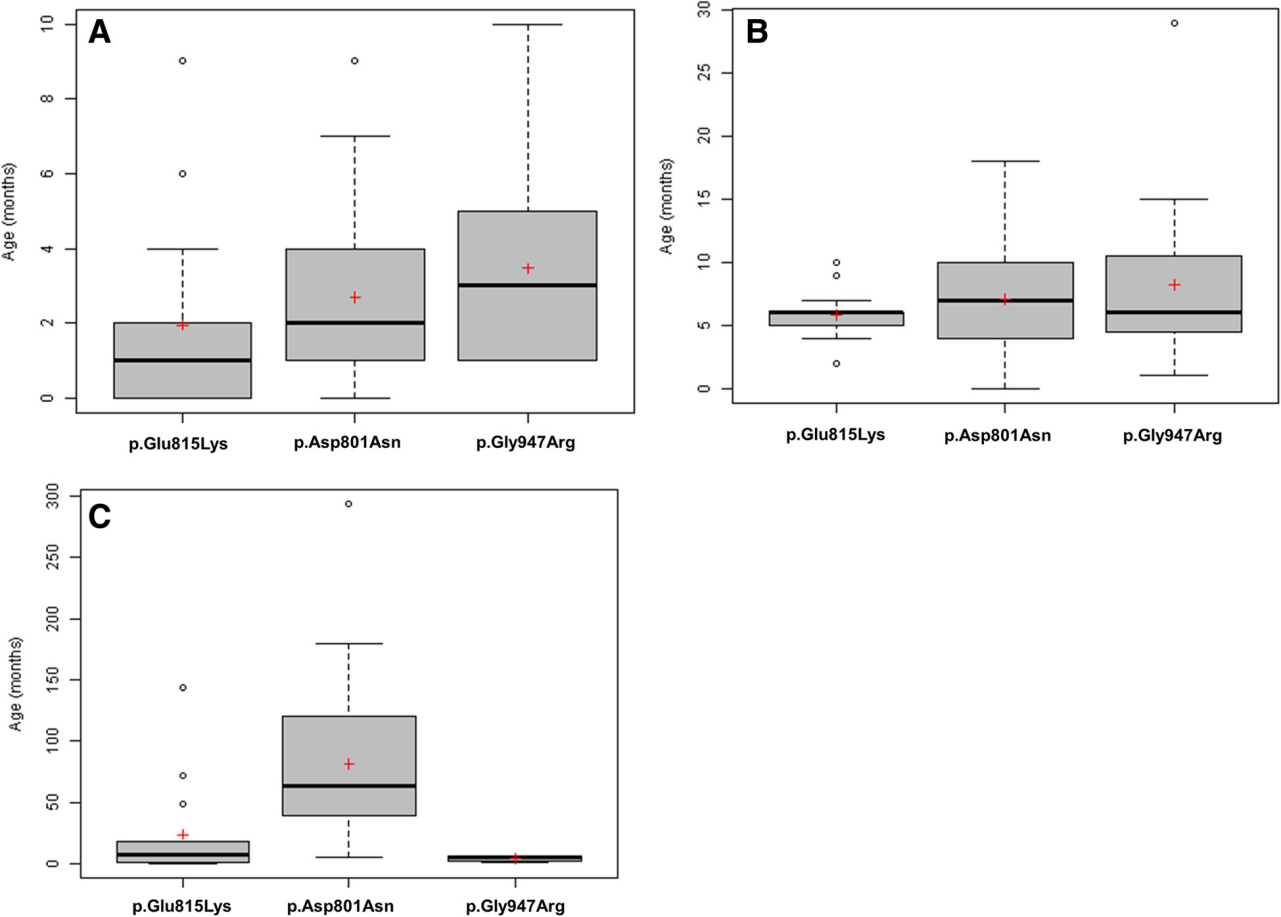
Distribution of age in months at: first paroxysmal event **a**, first plegic attack **b** and first epileptic seizures **c**. Black lines represent medians and the red crosses represent means. Some isolated values (very high or very low) are represented by circles

Patients with the p.Glu815Lys mutation tended to have an earlier age at the time of the first paroxysmal manifestation and first hemiplegic event, with frequent neonatal cases (Fig. 2a, b, Additional file 5). Relative to patients with p.Asp801Asn and p.Gly947Arg, they tended to have more frequent plegic attacks, but of shorter duration and less frequent dystonic attacks with a relatively short duration. Episodes of abnormal ocular movements occurred in almost the same percentage of patients with either of the three mutations.

p.Glu815Lys patients presented the most severe cognitive disability (*p* = 0.029), of whom half had severe and one third moderate intellectual disability. Likewise, 78 % presented moderate or severe language problems (age 6–12 years). During adulthood, none of the seven adult p.Glu815Lys patients were ever employed.

Patients with the p.Glu815Lys mutation also presented the greatest motor disability (Fig. 1). At an age between 6 and 12 years old, nearly half of them walked only with assistance and one third were wheelchair-bound (*p* = 0.039). They also appeared to demonstrate a higher degree of regression with regards to walking over time, compared to patients with either of the two other mutations, however, the period in which this occurred was variable, making a comparison difficult.

The majority of patients with either of the three most frequent mutations presented movement disorders over their lifetime; 72–89 % presented movement disorders between 6 and 12 years of age. More specifically, the majority (71 %) of p.Glu815Lys patients had dystonia at baseline (between paroxysmal events), and this was moderate to severe in 56 % (Fig. 1). However, only a third of them presented ataxia.

A greater proportion of patients with p.Glu815Lys presented epilepsy and status epilepticus, relative to patients with either p.Asp801Asn or p.Gly947Arg (Additional file 5). The onset of seizures occurred earlier in life for patients with p.Glu815Lys (often during the first year of life), relative to patients with p.Asp801Asn (*p* < 0.001) (Fig. 2c).

At age 6–12 years, a majority (78 %) of patients presented episodes of autonomic dysfunction and these patients presented more frequent attacks than patients with the other two mutations. This is speculated to be a precipitating factor for sudden death [9]. Four patients included in our cohort died; three had the p.Glu815Lys mutation and no mutation in the *ATP1A3* gene was reported in the fourth deceased patient.

#### p.Asp801Asn mutation

For patients with the p.Asp801Asn mutation*,* first paroxysmal and hemiplegic events occurred at an older age (Fig. 2a, b, Additional file 5). They had less frequent plegic attacks than the p.Glu815Lys group, but of longer duration and slightly more frequent tonic attacks (Fig. 1).

The majority (69 %) presented with moderate intellectual disability (*p* = 0.029) and 54 % had moderate or severe language problems (age 6–12 years). Among adult patients, one patient was independently employed and 25 % (eight patients) were working in an assisted environment. Behavioural disorders were more common in patients with the p.Asp801Asn mutation (in more than half the patients) compared to those with the other two mutations.

The majority (81 %) of p.Asp801Asn patients were able to walk independently at the age of 6–12 years (*p* = 0.039), but 63 % presented ataxia. Hence, there were fewer dystonic patients with p.Asp801Asn, in comparison to p.Glu815Lys mutation, and patients with p.Asp801Asn presented mainly mild dystonia (Fig. 1).

Fewer patients with p.Asp801Asn mutation presented epilepsy and status epilepticus, in comparison to the p.Glu815Lys group, and patients had rather infrequent seizures (Additional file 5). They also had an onset of seizures later in life (median 5 years), relative to patients with either p.Glu815Lys or p.Gly947Arg mutations (*p* < 0.001).

At age 6–12 years, the proportion of patients with episodes of autonomic dysfunction (44 %) was almost half that of p.Glu815Lys patients and similar to that of the p.Gly947Arg group.

#### p.Gly947Arg mutation

In this group, first events occurred at an even later age, compared to those with either p.Glu815Lys or p.Asp801Asn, with sometimes very late onset of plegic attacks (Fig. 2a, b, Additional file 5). Furthermore, p.Gly947Arg patients had the least frequent plegic attacks, but had a tendency to present more frequent and longer tonic attacks (Fig. 1).

None of the patients had severe intellectual disability and the majority (63 %) had only mild intellectual disability (*p* = 0 029). One of the five p.Gly947Arg adult patients was working in an assisted environment. At age 6–12 years, only 30 % of the p.Gly947Arg group presented moderate or severe language problems. Of note, a large proportion of patients, each with one of the three mutations, presented dysarthria that could further complicate verbal communication even in patients with mild intellectual disability.

All but one p.Gly947Arg patients (91 %) walked independently at the age 6–12 years (*p* = 0.039). The remaining patient was able to walk with help. At baseline, p.Gly947Arg patients appeared the least ataxic and/or dystonic, compared to the two other groups (Fig. 1).

Fewer patients harbouring the p.Gly947Arg mutation presented epilepsy compared to the other two groups (Additional file 5, Fig. 1). Surprisingly, the onset of seizures for epileptic subjects with p.Gly947Arg occurred earlier in life, relative to patients with the other two mutations (even earlier than for p.Glu815Lys mutation) (*p* < 0.001). Patients with p.Gly947Arg presented autonomic attacks to the same extent as those with p.Asp801Asn patients.

Finally ~15 % of the analysed patients were negative to the molecular analysis of *ATP1A3* gene. When the two groups were compared, no difference was observed regarding the frequency or length of plegic or tonic attacks, or the presence of abnormal ocular movements. Moreover, intellectual disability was similarly present in the majority of patients with and without *ATP1A3* mutations (Additional file 5).

No difference was reported with regards to the acquisition of gait and presence of abnormal movements. Similarly, the incidence of epilepsy and status epilepticus was comparable between the two groups.

The absence of statistically significant differences between the mutated and non-mutated patients can be in part explained by the small sample size (with in particular a small number of patients without mutations) and also by the correction of the multiplicity of tests.

#### Mutational clusters

**Fig. 3:**
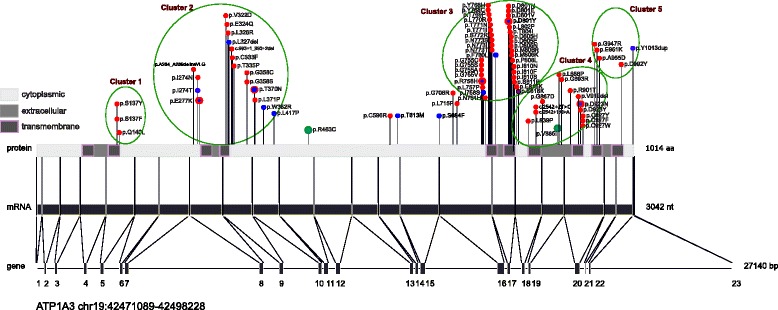
Location of mutations in *ATP1A3* gene, mRNA and protein. Numbers 1–23 represent gene exons; bp: base pairs; nt: nucleotides; aa: amino acids. AHC2 mutations are presented as red dots, RDP mutations as blue dots and two rare polymorphisms identified in the general population as green dots. The p.Glu818Lys mutation found in CAPOS families is shown as a purple dot. Mutations shared between AHC2 and RDP phenotypes are presented as red dots with a blue dot inside. The green circles represent the five mutational clusters that are located at the loops formed by an extracellular domain, the two adjacent transmembrane domains, and the surrounding regions of the cytoplasmic domain

Heinzen et al. [21] first recognized that nearly all AHC-causing *ATP1A3* mutations affect regions in or near transmembrane domains. Rosewich et al. [33] introduced the notion of mutational clusters. The observation of clustering was further confirmed in our cohort with the addition of new mutations. We have therefore further developed this notion within the *ATP1A3* gene, and outline five mutational clusters situated and corresponding to the loop formed by an extracellular domain, the two adjacent transmembrane domains, as well as the surrounding regions of the cytoplasmic domain (Fig. 3). The distribution of the novel mutations identified in our study, together with those previously reported, suggests that AHC2 and RDP are associated with similar areas of mutation clusters (Fig. 3). The clinical presentation of patients grouped according to these mutational clusters was investigated in order to establish whether different clusters could be correlated with particular phenotypes. Similarities in clinical phenotype were observed between patients belonging to the same mutational cluster (Table 1).

**Table 1:** Genotype—phenotype correlations

Genotype	Phenotype of patients encountered in this study										
	Number of patients	Age at onset: first event/first hemiplegic attack	Age at last observation/Gender	Intellectual disability	Walking problems	Ataxia	Dystonia	Hemiplegia/Double hemiplegia attacks	Tonic attacks	Epilepsy	Other patients’ references
**Cluster 1**											
p.Ser137Phe	1	Unknown	20y / F	++	Unknown	+	+	++++	++++	++	[21, 31]
p.Ser137Tyr	2	4 m / 4 m	9y / F	++	-	+	+	++++	Unknown	Remission	[21, 34, 35]
			2y / M	+	+		-	++++	-	-	
**Cluster 2**											
**p.Ala264_Ala289delinsValLeuGly**	1	2 m / 2 m	34y / M	-	++ (Regression- lost walking)	-	+++	+	+	-	
p.Ile274Asn	1	Neonatal / 33 m	10y / M	+	-	-	+	+++++	+	-	[21, 22, 31]
p.Glu324Gln	1	1 m / 5 m	16y / M	+++	++ (Regression)	-	+++	++++	++++	-	[31]
p.Leu326Arg	1	8 m / 15 m	4y / F	+	-	-	-	Remission	Remission	-	[31]
**c.993 + 1_993 + 2del** ^**$**^	1	12 m / 12 m	6y / M	-	-	-	+	+++	+	Remission	
p.Cys333Phe	1	1 m / 15 m	4y / M	+	+ (Regression)	-	+	++++	+++	Remission	[21, 31]
**p.Gly358Ser**	1	Neonatal / 6 years	11y / M	+++	+	+		+++++	++++	++++	
**Cytoplasmic domain between Cluster 2 and 3**											
p.Cys596Arg	1	1 m / 24 m	21y / M	+++	-	+	+	+++	+++	+	[31]
**p.Leu715Pro** ^**$**^	1	Neonatal / 3 m	2y / M	++	++	+	+	+++++	+	+++++	
**Cluster 3**											
p.Gly755Ser	3	4 m / 5 m	24y / M	-	-	Unknown	++	+++++	+++++	Remission	[21, 31, 33–35]
		6 m / 8 m	8y / M	+	-	++	Unknown	++++	++++	++	
		6 m / 6 m	8y / M	Unknown	-	+	-	++++	-	-	
p.Ser772Arg	2	9 m / 9 m	20y / M	++	+	+	++	+	+	+	[22, 31, 35]
		5 m / 10 m	7y / M	++	-	-	++	++++	++++	-	
p.Asn773Ser	2 mono-zygotic twins	Neonatal / 22 m	10y / F	+	-	-	-	++	+	Remission	[21]
		Neonatal / 22 m	10y / F	+	-	-	-	+++	++++	Remission	
p.Asp801Asn	57	2 m / 7 m (medians)	Median 15.7y	++	-	+++	++	++++	++++	+	[8, 21–23, 31, 33–35]
**p.Asp801Val** ^**$**^	1	21 m / 21 m	23y / M	-	-	++	+	Remission	++++	-	
p.Thr804Ile	2	2 m / 9 m	13y / F	+	-	-	+	++	++	-	[31, 33]
		5 m / 5 m	10y / F	++	-	+	-	++	++	-	
p.Met806Arg	1	13 m / 13 m	6y / M	+	-	-	+	+++	+++	Remission	[21]
p.Ile810Asn ^£^	1	7 m / 8 m	20y / M	++	-	+	-	+++	+++	Remission	[35]
p.Ser811Pro	2	1 m / 1 m	24y / F	++	+	++	++	+++++	+++++	++	[21, 31]
		1 m / 1 m	20y / F	++	++	+	++	++++	-	+	
p.Glu815Lys	22	1 m / 6 m (medians)	Median 7.8y	+++	++	++	+++	++++	++++	+++	[8, 21–23, 31, 33–35]
**Cluster 4**											
p.Leu839Pro	1	Neonatal	3 m / F	NA	NA	NA	++	+	++++	-	[31, 35]
c.2542 + 1G > A	1	1 m / 10 m	26y / F	++	+	-	+	+	-	+++	[21, 22, 31, 33]
**p.Leu888Pro** ^**$**^	1	1 m / 7 m	19y / M	+++	-	-	++	++	++	+++	
p.Val919del	2	Unknown	3y / F	+++	++		+	+++	+++	+	[21, 31]
		Neonatal / 5 m	17y / M	++	-	-	++	++++	++++	-	
p.Asp923Asn	2	4 m / 29 m	7y / F	-	-	+	-	+++	-	-	[34, 35, 40]
		11 m / 24 m	4y / F	+	-	-	+	++	-	-	
p.Cys927Phe	1	18 m / 18 m	15y / F	++	-	+	-	Remission	+++++	-	[34]
**p.Cys927Trp**	1	4 m / 4 m	37y / M	+	-	+	+	+	Remission	Remission	
**Cluster 5**											
p.Gly947Arg	15	3 m / 6 m (medians)	Median 15y	+	-	+	+	+++	+++	+	[8, 21, 31, 33–35]
p.Glu951Lys	1	4 m / 10 m	20y / M	++	+	+++	+	Remission	++	-	
p.Ala955Asp	1	Neonatal	4y / M	+++	++	-	+	+++++	++++	++	[21]
p.Asp992Tyr	1	4 m / 8 m	32y / M	+	Unknown	Unknown	+++	+++	++	+	[21, 34]

Novel mutations found in this study are given in bold characters. Of them, *de novo* mutations (both parents available and tested negative) are marked by the symbol $; Y: years, M: male, F: female, m: months; £: p.Ile810Asn (c.2429 T > A) corresponds to the Myshkin mice mutation; Remission, means no present at the last observation; First event: first paroxysmal event of the disease, either hemiplegic or other (i.e. abnormal ocular movements, double hemiplegia, tonic/dystonic attacks); Unknown: missed information; NA: not applicable because of young age at last observation. Reference sequences for corresponding ATP1A3 transcript and protein were [NM_152296.3] and [Uniprot P13637], respectively

### Interesting case reports within mutational clusters

We report a novel mutation in cluster 2, p.Ala264_Ala289delinsValLeuGly, identified in a 34-year-old man with no intellectual disability (the patient had a degree in graphic design), but who presented motor regression due to progressive disabling dystonia. Whereas he was experiencing bouts of hemiplegic/dystonic attacks in a typical AHC manner, he also presented a bi-phasic severe permanent deterioration of his dystonia after stressful events during adolescence (minor head trauma at first and subsequent orthopaedic surgery with complications). We believe this patient presents an intermediate AHC2/RDP phenotype (Table 1). This case appears even more interesting if we consider that this cluster harbours many mutations associated with RDP [21, 26, 27] (Fig. 3).

With the exception of the p.Glu815Lys mutation, mutations in cluster 3, especially those clustering at a location that corresponds to the transmembrane domain M6 (Fig. 3), are associated with mild-moderate phenotype, similar to p.Asp801Asn. Amino acid position 801 is a mutation hotspot and mutations occur at this position in both AHC2 (p.Asp801Asn, p.Asp801Glu, p.Asp801Tyr) [8, 21–23, 31, 33–35] and RDP (p.Asp801Tyr) [21, 26]. A novel p.Asp801Val mutation was found in a patient with a particularly mild phenotype, presenting a late onset of symptoms at 21 months, no intellectual disability, independent walking and at 23 years old, no more hemiplegic attacks and only weekly dystonic episodes.

In cluster 1 (Fig. 3, Table 1), the p.Ser137Tyr substitution was previously reported to yield a severe phenotype [34], in contrast to our report of two patients with no major disability.

The p.Ile274Asn mutation in cluster 2 was previously reported to be associated with an unusual phenotype first described in a familial case, in which the index patient presented with late-onset episodes at 3 years of age and mild intellectual disability [21]. The patient with p.Ile274Asn mutation in our cohort had similar characteristics. In the same cluster the p.Leu326Arg mutation was present in one patient with only mild symptoms that resolved with use of flunarizine, with remission of hemiplegic attacks. Whereas a patient with the c.993 + 1_993 + 2del mutation had no intellectual disability, another with the p.Cys333Phe mutation had mild, and another with the p.Gly358Ser mutation exhibited severe intellectual disability, although hemiplegic attacks began unusually late in life in the latter.

Regarding cluster 3 (Fig. 3, Table 1), three patients harboured the mutation p.Gly755Ser. All three presented a mild phenotype. This is in contrast to a previous report in which this mutation was associated with a severe phenotype [34]. The p.Ser772Arg mutation was previously reported in a child with normal intellect [22], contrasting with two cases in our study presenting moderate intellectual disability.

Amino acid 927 is a mutation hotspot in cluster 4. The p.Cys927Tyr and p.Cys927Phe mutations have previously been reported in patients with AHC [23, 34] and we identified a new mutation, p.Cys927Trp. The two patients harbouring the p.Cys927Phe and Cys927Trp mutations respectively had rare or no hemiplegic attacks with age.

The precise pathological mechanism resulting from *ATP1A3* mutations so far remains obscure. Amino acids 801 and 947 are located on the transmembrane domains M6 and M9, respectively, whereas amino acid 815 has an intracellular location. It is so far unclear what effect these mutations have on the α3 subunit, but based on preliminary studies [21], protein expression levels appear to be largely unaffected. Such mutations may therefore lead to hypomorphic effects which may influence ATPase activity. Weigand and colleagues [46] initially suggested that binding of the α3 subunit to ouabain may play a possible pathophysiological role. However, unlike the p.Asp801Asn mutation, both the p.Glu815Lys and p.Gly947Arg mutations prevent binding of the α3 subunit to ouabain, yet these latter mutations, according to our results, were associated with very different phenotypes. Thus, although the role of endogenous ouabain should further be investigated, it cannot explain differences in phenotype alone. A more recent study [47] attempted to explore the molecular pathological mechanisms concerning the three most frequent mutations. Authors suggested that loss of forward cycling function was unlikely to underlie the observed clinical heterogeneity in AHC, and the extent of dominant negativity was similar between p.Asp801Asn, p.Gly947Arg and p.Glu815Lys. But proton current amplitude was profoundly reduced in the mutation p.Glu815Lys compared to p.Asp801Asn and p.Gly947Arg mutations.

The large multinational sample of AHC patients included in our study provides a statistically strong confirmation of the rate of different *ATP1A3* mutations. Mutation in the *ATP1A3* gene was identified in 85 % patients (78–100 % in other series) [8, 21–23, 31, 33–35, 48], with the p.Asp801Asn, p.Glu815Lys and p.Gly947Arg mutations present in 43, 16 and 11 %, respectively (31–39 %, 20–23 % and 15 %, in other series) [33, 35, 48]. Overall, 34 different mutations were identified, of which 7 have not been described previously.

Within the limit of our present knowledge, we have defined distinct clinical profiles for patients harbouring each of the three most frequent mutations, with the most severe phenotypic expression associated with p.Glu815Lys, followed by p.Asp801Asn and lastly p.Gly947Arg. The more pronounced phenotypic expression associated with p.Glu815Lys, relative to other *ATP1A3* mutations, is in agreement with a recent study [31]. Previous studies in smaller cohorts of 35 and 51 patients, reported the severity of the p.Glu815Lys mutation concerning neonatal onset, motor disability and presence of status epilepticus and respiratory paralysis in the former [34] and a correlation with epilepsy in the latter [35]. The larger number of patients studied per mutation in this study provides a more comprehensive description of clinical profiles, allowing different clinical profiles to be compared.

We have further described a number of aspects of AHC that appear to be specific to certain mutations. Although some of the differences observed were not statistically significant, it should be emphasized that this may be due to the small number of patients with a given mutation, combined with the phenotypic complexity of the disorder. Indeed, when taken separately, the different major symptoms of AHC (such as epilepsy, movement disorders and cognition) are known to involve different neuronal networks, although these unavoidably interact. Such “symptoms” may even be considered as diseases *per se*. Each of these “major symptoms” may have their proper index of severity and it should be kept in mind that it is the combination of all these components that determines the severity of the AHC disorder as an entity. It could be hypothesized that a given mutation influences only one or more of these “major symptoms”, while sparing others. If this is the case, only studies with much larger cohorts may eventually better highlight the specific role of each mutation.

## Conclusion

Our study shows, based on a very extensive multinational cohort, that the phenotypic variation observed in AHC patients is mirrored in the heterogeneity of mutations affecting the *ATP1A3* gene. We have described the clinical profiles of patients harbouring the three most frequent mutations (p.Glu815Lys, p.Asp801Asn and p.Gly947Arg) and reported extensive clinical information for patients with less common mutations, by considering the different mutations within specific clusters. Our results support the notion that, although it is clear that the α3 subunit is implicated in the pathogenesis of AHC, the presence of individual variability in patients with the same mutation implies that other modifier genes or epigenetic factors play a role and this should be investigated in future studies.

## Appendix 1: The Italian IBAHC Consortium

Maria Teresa Bassi, Renato Borgatti, Roberta Cernetti, Gabriella Di Rosa, Filippo Franchini, Antonio Gambardella, Manlio Giacanelli, Melania Giannotta, Giuseppe Gobbi, Tiziana Granata, Elisa De Grandis, Renzo Guerrini, Fiorella Gurrieri, Gemma Incorpora, Nardo Nardocci, Giovanni Neri, Francesca Ragona, Margherita Santucci, Stefano Sartori, Michela Stagnaro, Danilo Tiziano, Rosaria Vavassori, Edvige Veneselli, Federico Vigevano, Claudio Zucca.

## Appendix 2: The French AHC Consortium

Aicardi J, An I, Arbues AS, Arzimanoglou A, Bahi-Buisson N, Barthez M-A, Billette de Villemeur T, Bourgeois M, Bru M, Chabrol B, Chaigne D, Chaunu MP, Chiron C, Cournelle AM, Davoine C-S, De St Martin A, Deny B, Desguerres I, Des Portes V, Doummar D, Dulac O, Dusser A, Gerard M, Gitiaux C, Godet Kiesel I, Gokben S (Turkey), Goutieres F, Guerrin M-H, Heron-Longe B, Hubsch-Bonneaud C, Hully M, Husson M, Ioos Ch, Kaminska A, Laroche C, Lazaro L, Lepine A, Magy L, Marchal C, Michel J, Milh M, Motte J, Moutard ML, Napuri S, Nassogne MC (Belgium), Neau JP, Nicole S, Panagiotakaki E, Passemard S, Pedespan JM, Penniello-Valette MJ, Poncelin D, Ponsot G, Poulat A-L, Pouplard F, Rabilloud M, Riant F, Rivier F, Roelens P, Roubergue A, Sanlaville D, Tardieu M, Veyrieres S.

## Appendix 3: The International AHC Consortium

Alexis Arzimanoglou (Scientific Coordinator), Rosaria Vavassori (Data Manager), Eleni Panagiotakaki (Node Coordinator, France), Elisa de Grandis (Node Coordinator Italy), Carmen Fons (Node Coordinator Spain), Sanjay Sisodiya (Node Coordinator UK), Peter de Jonghe (Node Coordinator Belgium-Antwerp), Christophe Goubeau (Node Coordinator Belgium – Leuven), Arn M.J.M. van den Maagdenberg (Node Coordinator Leiden - The Netherlands), Mohamad Mikati (Node Coordinator USA), Ingrid Scheffer (Node Coordinator Australia), Sona Nevsimalova (Node Coordinator Czech Republic) and members of national centers not already figuring in national Consortia: David Kemlink and Anna Krepelova (Czech Republic); Miriam Kolnikova and Pavol Sykora (Slovakia); Juan Kaski, Michael Hanna and Henry Houlden (UK); Adriana Ulate-Campos, Ramón Cancho, Jesús Eiris, Eduardo López-Laso and Ramón Velázquez (Spain), Ines Carilho (Portugal), Laurie Ozelius, Mount Sinai School of Medicine, (USA); Arvid Suls and Berten Ceulemans (Belgium - Antwerp); Gunnar Buyse and Michela di Michele (Belgium-Leuven); Michel Ferrari and Cacha M.P.C.D. Peeters-Scholte (The Netherlands – Leiden).

## Additional files

Additional file 1:
**Table of** ***ATP1A3*** **mutations (in this study and in previous studies).** (DOCX 31 kb) Additional file 2:
**Clinical Information included in the Questionnaire.** (DOCX 14 kb) Additional file 3:
**Primers used to sequence the ATP1A3 exons and adjacent splice sites.** (DOCX 18 kb) Additional file 4:
**Taqman-based genotyping assays used to establish unique patient identities.** (DOCX 17 kb) Additional file 5:
**Clinical phenotype of patients with the three most common mutations, and of patients without and with mutations, in the** ***ATP1A3*** **gene.** (DOCX 46 kb)

## Abbreviations

AHC: Aalternating hemiplegia of childhood
CAPOS syndrome: Cerebellar ataxia, areflexia, pes cavus, optic atrophy, sensorineural hearing loss
ENRAH: European Network for Research on Alternating Hemiplegia
IQ: Intellectual quotient
nEUroped: European Network on Rare Paediatric Neurological Diseases
RDP: Rapid-onset dystonia-parkinsonism
