# Supplementary material for: Clinical profile of patients with ATP1A3 mutations in Alternating Hemiplegia of Childhood—a study of 155 patients
Source: Orphanet J Rare Dis. 2015 Sep 26;10:123. doi: 10.1186/s13023-015-0335-5 (PMC4583741; doi:10.1186/s13023-015-0335-5)
Supplement: Additional file 1: — Table of ATP1A3 mutations (in this study and in previous studies). (DOCX 31 kb) [file 13023_2015_335_MOESM1_ESM.docx]

Additional File 1: Table of *ATP1A3* mutations (in this study and in previous studies).

| Nucleotide change  c.DNA | Amino acid change | Amino acid change (one letter code) | Location (exon, intron) | Type of mutation | SIFT | Polyphen-2 | Mutation Taster | AHC | RDP | CAPOS | Described in Reference (first publication) |
| --- | --- | --- | --- | --- | --- | --- | --- | --- | --- | --- | --- |
| **c.410C>T** | **p.Ser137Phe** | **p.S137F** | **Exon 5** | **missense** | **Deleterious** | **Probably damaging** | **Disease causing** | **x** |  |  | **21** |
| **c.410C>A** | **p.Ser137Tyr** | **p.S137Y** | **Exon 5** | **missense** | **Deleterious** | **Probably damaging** | **Disease causing** | **x** |  |  | **21** |
| c.419A>T | p.Gln140Leu | p.Q140L | Exon 5 | missense | Deleterious | Probably damaging | Disease causing | x |  |  | 21 |
| **c.791_866delinsTTCTGGG** | **p.Ala264_Ala289delinsValLeuGly** | **p.A264_A289delinsVLG** | **Exon 8** | **In-frame indel** | **-** | **-** | **-** | **x** |  |  | **Novel variant** |
| **c.821T>A** | **p.Ile274Asn** | **p.I274N** | **Exon 8** | **missense** | **Deleterious** | **Probably damaging** | **Disease causing** | **x** |  |  | **21** |
| c.821T>C | p.Ile274Thr | p.I274T | Exon 8 | missense | Deleterious | Probably damaging | Disease causing |  | x |  | 26 |
| c.829G>A | p.Glu277Lys | p.E277K | Exon 8 | missense | Deleterious | Probably damaging | Disease causing | x | x |  | 26 (RDP)  30 (AHC2) |
| c.965T>A | p.Val322Asp | p.V322D | Exon 8 | missense | Deleterious | Probably damaging | Disease causing | x |  |  | 22 |
| **c.970G>C** | **p.Glu324Gln** | **p.E324Q** | **Exon 8** | **missense** | **Deleterious** | **Probably damaging** | **Disease causing** | **x** |  |  | **31** |
| **c.977T>G** | **p.Leu326Arg** | **p.L326R** | **Exon 8** | **missense** | **Deleterious** | **Probably damaging** | **Disease causing** | **x** |  |  | **31** |
| c.979_981del | p.Leu327del | p.L327del | Exon 8 | In-frame deletion | - | - | - |  | x |  | 32 |
| **c.993+1_993+2del** |  |  | **Intron 8** | **abolition donor splice site** | **-** | **-** | **-** | **x** |  |  | **Novel variant (de novo)** |
| **c.998G>T** | **p.Cys333Phe** | **p.C333F** | **Exon 9** | **missense** | **Deleterious** | **Probably damaging** | **Disease causing** | **x** |  |  | **21** |
| c.1003A>C | p.Thr335Pro | p.T335P | Exon 9 | missense | Deleterious | Probably damaging | Disease causing | x |  |  | 33 |
| **c.1072G>A** | **p.Gly358Ser** | **p.G358S** | **Exon 9** | **missense** | **Deleterious** | **Probably damaging** | **Disease causing** | **x** |  |  | **Novel variant** |
| c.1072G>T | p.Gly358Cys | p.G358C | Exon 9 | missense | Deleterious | Probably damaging | Disease causing | x |  |  | 34 |
| c.1109C>A | p.Thr370Asn | p.T370N | Exon 9 | missense | Deleterious | Probably damaging | Disease causing | x | x |  | 33 (RDP)  35 (AHC2) |
| c.1112T>C | p.Leu371Pro | p.L371P | Exon 9 | missense | Deleterious | Probably damaging | Disease causing | x |  |  | 22 |
| c.1144T>C | p.Trp382Arg | p.W382R | Exon 9 | missense | Deleterious | Probably damaging | Disease causing |  | x |  | 33 |
| c.1250T>C | p.Leu417Pro | p.L417P | Exon 10 | missense | Deleterious | Probably damaging | Disease causing |  | x |  | 33 |
| **c.1786T>C** | **p.Cys596Arg** | **p.C596R** | **Exon 13** | **missense** | **Deleterious** | **Probably damaging** | **Disease causing** | **x** |  |  | **31** |
| c.1838C>T | p.Thr613Met | p.T613M | Exon 14 | missense | Deleterious | Probably damaging | Disease causing |  | x |  | 26 |
| c.2051C>T | p.Ser684Phe | p.S684F | Exon 15 | missense | Deleterious | Probably damaging | Disease causing |  | x |  | 36 |
| c.2116G>A | p.Gly706Arg | p.G706R | Exon 16 | missense | Deleterious | Probably damaging | Disease causing | x |  |  | 35 |
| **c.2144T>C** | **p.Leu715Pro** | **p.L715P** | **Exon 16** | **missense** | **Deleterious** | **Probably damaging** | **Disease causing** | **x** |  |  | **Novel variant (de novo)** |
| **c.2263G>A** | **p.Gly755Ser** | **p.G755S** | **Exon 16** | **missense** | **Deleterious** | **Probably damaging** | **Disease causing** | **x** |  |  | **21** |
| c.2263G>T | p.Gly755Cys | p.G755C | Exon 16 | missense | Deleterious | Probably damaging | Disease causing | x |  |  | 22 |
| c.2264G>C | p.Gly755Ala | p.G755A | Exon 17 | missense | Deleterious | Probably damaging | Disease causing | x |  |  | 34 |
| c.2264G>T | p.Gly755Val | p.G755V | Exon 17 | missense | Deleterious | Probably damaging | Disease causing | x |  |  | 31 |
| c.2267G>A | p.Arg756His | p.R756H | Exon 17 | missense | Deleterious | Probably damaging | Disease causing | x | x |  | 29 (RDP),  31 (AHC2) |
| c.2270T>C | p.Leu757Pro | p.L757P | Exon 17 | missense | Deleterious | Probably damaging | Disease causing | x |  |  | 33 |
| c.2273T>G | p.Ile758Ser | p.I758S | Exon 17 | missense | Deleterious | Probably damaging | Disease causing |  | x |  | 26 |
| c.2281A>C | p.Asn761His | p.N761H | Exon 17 | missense | Deleterious | Probably damaging | Disease causing | x |  |  | 31 |
| c.2302T>C | p.Tyr768His | p.Y768H | Exon 17 | missense | Deleterious | Probably damaging | Disease causing | x |  |  | 31 |
| c.2303A>G | p.Tyr768Cys | p.Y768C | Exon 17 | missense | Deleterious | Probably damaging | Disease causing | x |  |  | 31 |
| c.2305A>C | p.Thr769Pro | p.T769P | Exon 17 | missense | Deleterious | Probably damaging | Disease causing | x |  |  | 31 |
| c.2309T>G | p.Leu770Arg | p.L770R | Exon 17 | missense | Deleterious | Probably damaging | Disease causing | x |  |  | 35 |
| c.2312C>A | p.Thr771Asn | p.T771N | Exon 17 | missense | Deleterious | Benign | Disease causing | x |  |  | 34 |
| c.2312C>T | p.Thr771Ile | p.T771I | Exon 17 | missense | Deleterious | Benign | Disease causing | x |  |  | 35 |
| **c.2314A>C** | **p.Ser772Arg** | **p.S772R** | **Exon 17** | **missense** | **Deleterious** | **Probably damaging** | **Disease causing** | **x** |  |  | **31** |
| **c.2316C>G** | **p.Ser772Arg** | **p.S772R** | **Exon 17** | **missense** | **Deleterious** | **Probably damaging** | **Disease causing** | **x** |  |  | **35** |
| c.2316C>A | p.Ser772Arg | p.S772R | Exon 17 | missense | Deleterious | Probably damaging | Disease causing | x |  |  | 22 |
| **c.2318A>G** | **p.Asn773Ser** | **p.N773S** | **Exon 17** | **missense** | **Deleterious** | **Possibly damaging** | **Disease causing** | **x** |  |  | **21** |
| c.2318A>T | p.Asn773Ile | p.N773I | Exon 17 | missense | Deleterious | Probably damaging | Disease causing | X |  |  | 22 |
| c.2318A>C | p.Asn773Thr | p.N773T | Exon 17 | missense | Deleterious | Possibly damaging | Disease causing | x |  |  | 31 |
| c.2338T>C | p.Phe780Leu | p.F780L | Exon 17 | missense | Deleterious | Benign | Disease causing |  | x |  | 26 |
| **c.2401G>A** | **p.Asp801Asn** | **p.D801N** | **Exon 17** | **missense** | **Deleterious** | **Probably damaging** | **Disease causing** | **x** |  |  | **21** |
| **c.2402A>T** | **p.Asp801Val** | **p.D801V** | **Exon 17** | **missense** | **Deleterious** | **Probably damaging** | **Disease causing** | **x** |  |  | **Novel variant (de novo)** |
| c.2403T>C | p.Asp801Glu | p.D801E | Exon 17 | missense |  |  |  | x |  |  | 8 |
| c.2401G>T | p.Asp801Tyr | p.D801Y | Exon 17 | missense | Deleterious | Probably damaging | Disease causing | x | x |  | 26 (RDP),  31 (AHC2) |
| c.2405T>C | p.Leu802Pro | p.L802P | Exon 17 | missense | Deleterious | Probably damaging | Disease causing | x |  |  | 35 |
| **c.2411C>T** | **p.Thr804Ile** | **p.T804I** | **Exon 17** | **missense** | **Deleterious** | **Probably damaging** | **Disease causing** | **x** |  |  | **33** |
| c.2413G>C | p.Asp805His | p.D805H | Exon 17 | missense | Deleterious | Probably damaging | Disease causing | x |  |  | 35 |
| c.2413G>A | p.Asp805Asn | p.D805N | Exon 17 | missense | Deleterious | Probably damaging | Disease causing | x |  |  | 31 |
| c.2415C>G | p.Asp805Glu | p.D805E | Exon 17 | missense | Deleterious | Benign | Disease causing | x |  |  | 33 |
| **c.2417T>G** | **p.Met806Arg** | **p.M806R** | **Exon 17** | **missense** | **Deleterious** | **Possibly damaging** | **Disease causing** | **x** |  |  | **21** |
| c.2417T>A | p.Met806Lys | p.M806K | Exon 18 | missense | Deleterious | Benign | Disease causing | x |  |  | 35 |
| c.2423C>T | p.Pro808Leu | p.P808L | Exon 18 | missense | Deleterious | Probably damaging | Disease causing | x |  |  | 35 |
| c.2429T>G | p.Ile810Ser | p.I810S | Exon 18 | missense | Deleterious | Probably damaging | Disease causing | x |  |  | 21 |
| c.2428A>T | p.Ile810Phe | p.I810F | Exon 18 | missense | Deleterious | Probably damaging | Disease causing | x |  |  | 33 |
| **c.2429T>A** | **p.Ile810Asn** | **p.I810N** | **Exon 18** | **missense** | **Deleterious** | **Probably damaging** | **Disease causing** | **x** |  |  | **35** |
| **c.2431T>C** | **p.Ser811Pro** | **p.S811P** | **Exon 18** | **missense** | **Deleterious** | **Probably damaging** | **Disease causing** | **x** |  |  | **21** |
| **c.2443G>A** | **p.Glu815Lys** | **p.E815K** | **Exon 18** | **missense** | **Deleterious** | **Probably damaging** | **Disease causing** | **x** |  |  | **21** |
| c.2452G>A | p.Glu818Lys | p.E818K | Exon 18 | missense | Deleterious | Probably damaging | Disease causing | x |  | x | 28 (CAPOS)  37 (AHC2) |
| **c.2516T>C** | **p.Leu839Pro** | **p.L839P** | **Exon 18** | **missense** | **Deleterious** | **Probably damaging** | **Disease causing** | **x** |  |  | **35** |
| **c.2542+1:G>A** |  |  | **Intron 18** | **abolition donor splice site** | **-** | **-** | **-** | **x** |  |  | **21** |
| c.2542+2T>C |  |  | Intron 18 | Abolition donor splice site | - | - | - | x |  |  | 31 |
| c.2600G>A | p.Gly867Asp | p.G867D | Exon 19 | missense | Deleterious | Probably damaging | Disease causing | x |  |  | 38 |
| **c.2663T>C** | **p.Leu888Pro** | **p.L888P** | **Exon 19** | **missense** | **Deleterious** | **Benign** | **Disease causing** | **x** |  |  | **Novel variant (de novo)** |
| c.2677G>A | p.Gly893Arg | p.G893R | Exon 19 | missense | Deleterious | Probably damaging | Disease causing | x |  |  | 35 |
| c.2702G>C | p.Arg901Thr | p.R901T | Exon 20 | missense | Deleterious | Probably damaging | Disease causing | x |  |  | 31 |
| **c.2755_2757del** | **p.Val919del** | **p.V919del** | **Exon 20** | **In-frame deletion** | **-** | **-** | **-** | **x** |  |  | **21** |
| c.2767G>T | p.Asp923Tyr | p.D923Y | Exon 20 | missense | Deleterious | Probably damaging | Disease causing | x |  |  | 22 |
| **c.2767G>A** | **p.Asp923Asn** | **p.D923N** | **Exon 20** | **missense** | **Tolerated** | **Probably damaging** | **Disease causing** | **x** | **x** |  | **39 (RDP)**  **40 (AHC2)** |
| c.2780G>A | p.Cys927Tyr | p.C927Y | Exon 20 | missense | Deleterious | Probably damaging | Disease causing | x |  |  | 23 |
| **c.2780G>T** | **p.Cys927Phe** | **p.C927F** | **Exon 20** | **missense** | **Deleterious** | **Probably damaging** | **Disease causing** | **x** |  |  | **34** |
| **c.2781C>G** | **p.Cys927Trp** | **p.C927W** | **Exon 20** | **missense** | **Deleterious** | **Probably damaging** | **Disease causing** | **x** |  |  | **Novel Variant** |
| **c.2839G>A** | **p.Gly947Arg** | **p.G947R** | **Exon 21** | **missense** | **Deleterious** | **Probably damaging** | **Disease causing** | **x** |  |  | **21** |
| **c.2839G>C** | **p.Gly947Arg** | **p.G947R** | **Exon 21** | **missense** | **Deleterious** | **Probably damaging** | **Disease causing** | **x** |  |  | **21** |
| **c.2851G>A** | **p.Glu951Lys** | **p.E951K** | **Exon 21** | **missense** | **Deleterious** | **Probably damaging** | **Disease causing** | **x** |  |  | **31** |
| **c.2864C>A** | **p.Ala955Asp** | **p.A955D** | **Exon 21** | **missense** | **Deleterious** | **Probably damaging** | **Disease causing** | **x** |  |  | **21** |
| **c.2974G>T** | **p.Asp992Tyr** | **p.D992Y** | **Exon 22** | **missense** | **Deleterious** | **Probably damaging** | **Disease causing** | **x** |  |  | **21** |
| c.3038_3040dup | p.Tyr1013dup | p.Y1013dup | Exon 23 | In-frame duplication | - | - | - |  | x |  | 41 |

Mutations described in this study are marked in bold characters.

Only the first reference corresponding to each mutation is reported. If the same mutation has been identified in two diseases, the first reference corresponding to each disease is reported.
