# Supplementary material for: Clinical profile of patients with ATP1A3 mutations in Alternating Hemiplegia of Childhood—a study of 155 patients
Source: Orphanet J Rare Dis. 2015 Sep 26;10:123. doi: 10.1186/s13023-015-0335-5 (PMC4583741; doi:10.1186/s13023-015-0335-5)
Supplement: Additional file 2: — Clinical Information included in the Questionnaire. (DOCX 14 kb) [file 13023_2015_335_MOESM2_ESM.docx]

**Additional File 2 : Clinical Information included in the Questionnaire**

**GENERAL INFORMATION**

Patient Identifier / Gender / Birth Date / Date of inclusion to the database / Date of the diagnosis / Pregnancy / Delivery / Apgar / Age of Death / Age of first paroxysmal event / Type of first paroxysmal event / Age of first plegic attack / Age of definitely epileptic seizures / Migraine in the Family / Epilepsy in the Family / Other neurological disorders in the family / Kinship (proband, other)

**PAROXYSMAL EVENTS during lifetime**

Premonitory signs or aura / Tonic/(hemi)dystonic attacks / (hemi) plegic attacks / Plegic attacks affecting both sides / Abnormal ocular movements / Seizures-Epilepsy / Status epilepticus / Age of first Status Epilepticus / Headache / Autonomic Dysfunction / Disappearance of symptoms with sleep and immediately following sleep

**NON PAROXYSMAL FEATURES during lifetime**

Ataxia / Dysarthria / Pyramidal signs / Dystonia / Complex-other Movement Disorder / Muscle Tone / Intellectual Disability / Communication disorders / Behavioural disorders / Educational placement / Working employment / Details about Language Use

**FOLLOW-UP AT INCLUSION**

**PAROXYSMAL EVENTS**

**TONIC OR HEMI-DYSTONIC ATTACKS // PLEGIC ATTACKS**

Semiology / Impairment of Consciousness / Frequency / Length / Trigger events / Type of Trigger Events

**ABNORMAL OCULAR MOVEMENTS**

Semiology / Are they linked to another paroxysmal event?

**SEIZURES**

Semiology (focal, with or without secondary generalization; generalized; focal and generalized; unknown) / Frequency without / with treatment or drug / Is it linked in time to another paroxysmal event?

**STATUS EPILEPTICUS**

Semiology (convulsive; not convulsive; both; unknown) / Frequency

**HEADACHE**

Frequency / Is it linked to other paroxysmal event?

**EPISODES OF AUTONOMIC DYSFUNCTION (Including breathing difficulties)**

Frequency / Is it linked to other paroxysmal event?

**NON PAROXYSMAL FEATURES**

Walking (autonomous; with help; not possible; not applicable; unknown) / Ataxia / Pyramidal signs / Dystonia (outside of hemidystonic episodes) / Complex-other Movement Disorder / Muscle Tone / Dysarthria / Intellectual Disability (no; yes, mild; yes, moderate; yes, severe; not applicable; unknown) / Verbal Communication Disorder / Behavioural Disorders

**FOLLOW-UP AT 6-12 YEARS**

**(same questions as for follow-up at inclusion)**
