# Supplementary material for: Clinical profile of patients with ATP1A3 mutations in Alternating Hemiplegia of Childhood—a study of 155 patients
Source: Orphanet J Rare Dis. 2015 Sep 26;10:123. doi: 10.1186/s13023-015-0335-5 (PMC4583741; doi:10.1186/s13023-015-0335-5)
Supplement: Additional file 3: — Primers used to sequence the ATP1A3 exons and adjacent splice sites. (DOCX 18 kb) [file 13023_2015_335_MOESM3_ESM.docx]

**Additional File3: Primers used to sequence the ATP1A3 exons and adjacent splice sites.**

| **ATP1A3 EXON** | **Forward Primer Sequence:** | **Reverse Primer Sequence:** |
| --- | --- | --- |
| ATP1A3-EXON1 | agaggctcccagcccaag | cccacgaccacatggatt |
| ATP1A3-EXONS2-4 | ctcagagacacacagaacca | tgtgacactcactctgggta |
| ATP1A3-EXONS5-6 | taaggatctccgaaaggtg | cgacagacccttagattcaa |
| ATP1A3-EXON7 | acccaggcttctagctgtga | tccacacagttggtggaaaa |
| ATP1A3-EXON8 | cacctttcggagatcctta | gagcgtgcacttcttaattt |
| ATP1A3-EXONS9-11 | tgtctctgccctgtttctat | ctcccaaagttctggtgtta |
| ATP1A3-EXON12 | acagagcggacaggaatgag | ctttgggcagcatcacaac |
| ATP1A3-EXON13 | gacatagacagagcggacag | ataaaataaaggctgggatg |
| ATP1A3-EXON14 | acttcaccacggacaacctc | tcccagaaagaatgggacag |
| ATP1A3-EXON15 | cccaaagtccttcctcaggt | gtgaggacccaggagtcaag |
| ATP1A3-EXON16 | ttggggacctgaacttcta | tttaaaaagcctccaagtca |
| ATP1A3-EXON17 | agatcgcaccactgcactc | tcttcatgatgtcgctttcg |
| ATP1A3-EXON18 | agcgagactctgtctcaaaa | ctaggccacctaaacatcat |
| ATP1A3-EXON19 | ggcatactcccctctccaag | gatcttacggtgggcagaga |
| ATP1A3-EXON20 | gctgcagtgccactaactga | agagtgagaccctgcctcaa |
| ATP1A3-EXON21 | gcatgtctccccatctctgt | cctggggtcttcggagtaat |
| ATP1A3-EXON22 | tccagtcccctgaaactctg | cggagagatgggaagagaga |
| ATP1A3-EXON23 | cttctcacgggtctctgtctg | cccccagaatacaaaattgg |

Reference sequence for corresponding *ATP1A3* transcript was [NM_152296.3].
