# Supplementary material for: Clinical profile of patients with ATP1A3 mutations in Alternating Hemiplegia of Childhood—a study of 155 patients
Source: Orphanet J Rare Dis. 2015 Sep 26;10:123. doi: 10.1186/s13023-015-0335-5 (PMC4583741; doi:10.1186/s13023-015-0335-5)
Supplement: Additional file 4: — Taqman-based genotyping assays used to establish unique patient identities. (DOCX 17 kb) [file 13023_2015_335_MOESM4_ESM.docx]

**Additional File 4. Taqman-based genotyping assays used to establish unique patient identities.**

| **rs number** | **Assay ID** |
| --- | --- |
| rs2290682 | C__11709675_20 |
| rs1159148 | C__11908548_10 |
| rs2071856 | C__15869887_10 |
| rs6572635 | C__29157477_10 |
| rs4796555 | C__27941170_20 |
| rs11211328 | C__26787433_20 |
| rs2290987 | C__22273232_10 |
| rs2075960 | C___2990199_1_ |
| rs10255061 | C__29720817_10 |
| rs13094003 | C____473627_10 |
| rs5952451 | C__30106921_10 |
| rs5931272 | C__29560051_20 |
| rs6644970 | C__30273591_20 |
