# Supplementary material for: Clinical profile of patients with ATP1A3 mutations in Alternating Hemiplegia of Childhood—a study of 155 patients
Source: Orphanet J Rare Dis. 2015 Sep 26;10:123. doi: 10.1186/s13023-015-0335-5 (PMC4583741; doi:10.1186/s13023-015-0335-5)
Supplement: Additional file 5: — Clinical phenotype of patients with the three most common mutations, and of patients without and with mutations, in the ATP1A3 gene. (DOCX 46 kb) [file 13023_2015_335_MOESM5_ESM.docx]

Additional File 5: Clinical phenotype of patients with the three most common mutations, and of patients without, and with mutations, in the *ATP1A3* gene.

| **Clinical phenotype** | **p.Glu815Lys** | **p.Asp801Asn** | **p.Gly947Arg** | ***ATP1A3* negative** | ***ATP1A3* positive** |
| --- | --- | --- | --- | --- | --- |
| Number of patients percentage | 16%  (22/132 mutated patients) | 43%  (57/132 mutated patients) | 11%  (15/132 mutated patients) | 15%  (23/155 total patients) | 85%  (132/155 total patients) |
| **AGE AT ONSET** | | | | | |
| Age of first paroxysmal episode in months  (median-mean) **See (Fig. 2)** (min-max) | n=21  1 – 1.9  0 – 9 | n=56  2 – 2.7  0 – 9 | n=15  3 – 3.4  1 – 10 | n=23  3 – 5.8  0 – 24 | n=128  2 – 3  0 – 21 |
| Age of first hemiplegic episode in months  (median-mean) **See (Fig. 2)** (min-max) | n=21  6 - 5.8  2 – 10 | n=53  7 – 7  0 - 18 | n=15  6 - 8.2  1 – 29 | n=23  12 – 11  0 – 24 | n=123  6 – 8.5  0 – 72 |
| Type of first paroxysmal episode  Abnormal ocular movements  Hemiplegic or bilateral plegic  Tonic/Dystonic attacks | n=19  42% 8  21% 4  16% 3 | n=51  27% 14  25% 13  33% 17 | n=13  38% 5  0% 0  30% 4 | n=19  5% 1  26% 5  26% 5 | n=119  34% 41  21% 25  23% 28 |
| **PAROXYSMAL EVENTS (OTHER THAN EPILEPSY)** | | | | | |
| Hemiplegic episodes (6-12 years) | 100% (14/14) | 94% (36/38) | 91% (10/11) | 94% (16/17) | 95% (100/105) |
| Frequency hemiplegic episodes 6-12 y  NO  <=1/year  **See (Fig. 1)**  1-6/year  Monthly  Weekly  Daily | n=12  0% 0  0% 0  25% 3  8% 1  50% 6  16% 2 | n=38  5% 2  0% 0  5% 2  31% 12  44% 17  13% 5 | n=11  9% 1  0% 0  27% 3  36% 4  27% 3  0% 0 | n=16  6% 1  6% 1  12% 2  25% 4  37% 6  12% 2 | n=85  5% 4  1% 1  16% 14  28% 24  38% 33  10% 9 |
| Length hemiplegic episodes 6-12 y  NO  <=1 hour  **See (Fig. 1)**  1 – 6 hours  6 – 12 hours  12 – 24 hours  >=24 hours | n=14  0% 0  21% 3  42% 6  7% 1  7% 1  21% 3 | n=37  5% 2  21% 8  13% 5  11% 4  11% 4  38% 14 | n=11  9% 1  27% 3  9% 1  0% 0  9% 1  45% 5 | n=16  6% 1  31% 5  18% 3  6% 1  6% 1  31% 5 | n=86  4% 4  26% 23  21% 18  7% 6  10% 9  30% 26 |
| Dystonic episodes (6-12 years) | 71% (10/14) | 74% (26/35) | 82% (9/11) | 70% (12/17) | 77% (66/85) |
| Frequency dystonic episodes 6-12 y  NO  <=1/year  **See (Fig. 1)** 1-6/year  Monthly  Weekly  Daily | n=13  31% 4  15% 2  7% 1  7% 1  23% 3  15% 2 | n=31  29% 9  3% 1  6% 2  19% 6  29% 9  13% 4 | n=11  18% 2  9% 1  0% 0  36% 4  27% 3  9% 1 | n=16  31% 5  6% 1  12% 2  12% 2  25% 4  12% 2 | n=77  24% 19  5% 4  9% 7  21% 16  28% 22  11% 9 |
| Length dystonic episodes 6-12 y  NO  <=1 hour  **See (Fig. 1)** 1 – 6 hours  6 – 12 hours  12 – 24 hours  >=24 hours | n=14  28% 4  43% 6  7% 1  7% 1  7% 1  7% 1 | n=31  29% 9  42% 13  9% 3  3% 1  0% 0  16% 5 | n=10  20% 2  30% 3  10% 1  10% 1  10% 1  20% 2 | n=16  31% 5  37% 6  6% 1  6% 1  6% 1  12% 2 | n=78  24% 19  45% 35  13% 10  4% 3  2% 2  11% 9 |
| Abnormal Ocular Movements during lifetime | 100% (22/22) | 98% (55/56) | 93% (14/15) | 82% (19/23) | 98% (127/129) |
| Abnormal Ocular Movements (6-12 years) | 57% (8/14) | 58% (21/36) | 45% (5/11) | 59% (10/17) | 56% (47/83) |
| **COGNITION - BEHAVIOUR** | | | | | |
| Intellectual Disability during lifetime | 100% (22/22) | 98% (56/57) | 100% (15/15) | 91% (20/22) | 95% (124/130) |
| Intellectual Disability (6-12 years) | 100% (14/14) | 100% (36/36) | 100% (11/11) | 100% (13/13) | 95% (82/86) |
| Degree of Intellectual Disability (6-12 years):  NO  **See (Fig. 1)** Mild  Moderate  Severe | n=14  0% 0  21% 3  28% 4  50% 7 | n=36  0% 0  22% 8  69% 25  8% 3 | n=11  0% 0  63% 7  36% 4  0% 0 | n=13  0% 0  23% 3  77% 10  0% 0 | n=86  4% 4  32% 28  46% 40  16% 14 |
| Verbal Communication Disorder during lifetime | 47% (10/21) | 58% (32/55) | 43% (6/14) | 57% (12/21) | 53% (66/123) |
| Verbal Communication Disorder (6-12 years) | 93% (13/14) | 79% (26/33) | 70% (7/10) | 71% (10/14) | 80% (64/80) |
| Degree of Verbal Communication Disorder (6-12 years):  NO  **See (Fig. 1)** Mild  Moderate  Severe | n=14  7% 1  14% 2  35% 5  43% 6 | n=33  21% 7  24% 8  30% 10  24% 8 | n=10  30% 3  40% 4  20% 2  10% 1 | n=14  28% 4  28% 4  28% 4  14% 2 | n=80  20% 16  26% 21  30% 24  24% 19 |
| Dysarthria (6-12 years) | 80% (4/5) | 92% (23/25) | 100% (8/8) | 63% (7/11) | 86% (50/58) |
| Behavioral Troubles (during lifetime) | 47% (10/21) | 58% (32/55) | 43% (6/14) | 57% (12/21) | 53% (66/123) |
| Behavioral Troubles present at inclusion | 40% (8/20) | 52% (26/50) | 27% (3/11) | 47% (10/21) | 45% (52/114) |
| Behavioral Troubles 6-12 y | 43% (6/14) | 58% (21/36) | 9% (1/11) | 64% (9/14) | 49% (40/82) |
| **MOTOR DISABILITY** | | | | | |
| Walking (6-12 years)  Autonomous  **See (Fig. 1)** With Help  Not possible | n=14  28% 4  43% 6  28% 4 | n=37  81% 30  13% 5  5% 2 | n=11  91% 10  9% 1  0% 0 | n=17  76% 13  17% 3  6% 1 | n=87  74% 65  16% 4  9% 8 |
| Ataxia during lifetime | 41% (7/17) | 71% (38/53) | 57% (8/14) | 37% (7/19) | 60% (70/116) |
| Ataxia 6-12 y | 33% (3/9) | 63% (21/33) | 22% (2/9) | 38% (5/13) | 48% (35/72) |
| Degree of ataxia 6-12 y  NO  **See (Fig. 1)** Mild  Moderate  Severe | n=9  66% 6  22% 2  0% 0  11% 1 | n=33  36% 12  30% 10  21% 7  12% 4 | n=9  78% 7  22% 2  0% 0  0% 0 | n=13  61% 8  23% 3  7% 1  7% 1 | n=72  51% 37  26% 19  14% 10  8% 6 |
| Dystonia during lifetime | 68% (15/22) | 73% (42/57) | 86% (13/15) | 57% (12/21) | 74% (96/129) |
| Dystonia 6-12 y | 71% (10/14) | 63% (22/35) | 54% (6/11) | 56% (9/16) | 62% (52/84) |
| Degree of dystonia 6-12 y  NO  **See (Fig. 1)** Mild  Moderate  Severe | n=14  28% 4  14% 2  35% 5  21% 3 | n=35  37% 13  43% 15  17% 6  3% 1 | n=11  45% 5  45% 5  9% 1  0% 0 | n=16  44% 7  31% 5  12% 2  12% 2 | n=84  38% 32  37% 31  19% 16  6% 5 |
| Other/Complex movement disorder during lifetime | 68% (15/22) | 52% (29/56) | 80% (12/15) | 62% (13/21) | 60% (77/127) |
| Other/Complex movement disorder 6-12 y | 69% (9/13) | 40% (14/35) | 54% (6/11) | 68% (11/16) | 47% (39/82) |
| Movement disorders (all) during lifetime | 100% (20/20) | 94% (52/55) | 100% (15/15) | 82% (18/22) | 95% (121/127) |
| Movement disorders 6-12 y | 78% (11/14) | 89% (33/37) | 72% (8/11) | 87% (14/16) | 84% (73/87) |
| **EPILEPSY** | | | | | |
| Epilepsy during lifetime | 82% (18/22) | 55% (31/56) | 40% (6/15) | 50% (11/22) | 59% (76/129) |
| Age of onset of first seizure in months  (median-mean)  **See (Fig. 2)** (min-max) | n=14  7,5 - 23  0 - 144 | n=26  63 - 81  5 - 294 | n=5  4- 5  1 - 6 | n=7  2 – 14  0 – 84 | n=63  21 – 53  0 – 294 |
| Epilepsy present at inclusion | 47% (10/21) | 30% (17/56) | 13% (2/15) | 24% (5/21) | 32% (42/129) |
| Epilepsy at 6-12 years | 50% (7/14) | 39% (14/36) | 18% (2/11) | 23% (4/17) | 35% (30/86) |
| Semiology of seizures at 6-12 years  FOCAL, +/- SECONDARY GENERALISATION  GENERALIZED  FOCAL AND GENERALIZED | n=7  71% 5  0% 0  28% 2 | n=13  61% 8  30% 4  7% 1 | n=2  50% 1  0% 0  50% 1 | n=4  75% 3  25% 1  0% 0 | n=29  58% 17  27% 8  14% 4 |
| Frequency of seizures at 6-12 years  <=1/year  **See (Fig. 1)** 1-6/year  Monthly  Weekly | n=6  0% 0  33% 2  50% 3  16% 1 | n=14  50% 7  43% 6  7% 1  0% 0 | n=2  0% 0  100% 2  0% 0  0% 0 | n=3  100% 3  0% 0  0% 0  0% 0 | n=29  34% 10  41% 12  14% 4  10% 3 |
| Status epilepticus during lifetime | 38% (8/21) | 21% (12/56) | 20% (3/15) | 19% (4/21) | 24% (31/127) |
| Age of onset of first status in months  (median-mean) . (min-max) | n=2  114- 114  0-228 | n=6  60 - 81  39 - 152 | n=3  180 - 190  20 - 370 | n=3  9 - 41  7 - 108 | n=16  97 - 119  0 - 370 |
| Status epilepticus present at inclusion | 24% (5/21) | 12% (7/57) | 15% (2/13) | 5% (1/20) | 14% (18/128) |
| Status epilepticus at 6-12 years | 21% (3/14) | 11% (4/37) | 9% (1/11) | 0% (0/17) | 11% (10/87) |
| Semiology of status at 6-12 years  CONVULSIVE  **See (Fig. 1)** NOT CONVULSIVE  BOTH | n=3  66% 2  33% 1  0% 0 | n=4  100% 4  0% 0  0% 0 | n=0  0% 0  0% 0  0% 0 | n=0  0% 0  0% 0  0% 0 | n=9  78% 7  22% 2  0% 0 |
| **AUTONOMIC DYSFUNCTION** | | | | | |
| Autonomic dysfunction during lifetime | 95% (21/22) | 74% (37/50) | 71% (10/14) | 77% (17/22) | 76% (89/117) |
| Autonomic dysfunction present at inclusion | 62% (13/21) | 44% (23/52) | 43% (6/14) | 62% (13/21) | 45% (54/120) |
| Autonomic dysfunction 6-12 y | 78% (11/14) | 44% (15/34) | 50% (5/10) | 35% (5/14) | 52% (42/81) |
| Frequency episodes autonomic dysfunction 6-12 y  NO  <=1/year  **See (Fig. 1)**  1-6/year  Monthly  Weekly  Daily | n=12  25% 3  0% 0  8% 1  41% 5  8% 1  16% 2 | n=29  65% 19  3% 1  10% 3  7% 2  14% 4  0% 0 | n=9  55% 5  11% 1  11% 1  0% 0  11% 1  11% 1 | n=18  44% 8  0% 0  11% 2  5% 1  28% 5  11% 2 | n=106  62% 66  3% 3  6% 7  11% 12  13% 14  4% 4 |
|  | | | | | |
